# Assessing Associations of Three Types of Higher‐Risk Medication Use and Mortality in Older Adults

**DOI:** 10.1111/jgs.70569

**Published:** 2026-07-08

**Authors:** Alexander Chaitoff, Katharina Tabea Jungo, Lillian Min, Donovan T. Maust

**Affiliations:** ^1^ Department of Internal Medicine University of Michigan School of Medicine Ann Arbor Michigan USA; ^2^ Center for Clinical Management Research VA Ann Arbor Healthcare System Ann Arbor Michigan USA; ^3^ Institute of Primary Health Care (BIHAM), University of Bern Bern Switzerland; ^4^ Center for Healthcare Delivery Sciences (C4HDS), division of Pharmacoepidemiology and Pharmacoeconomics, Department of Medicine Brigham and Women's Hospital and Harvard Medical School Boston Massachusetts USA; ^5^ Department of Psychiatry University of Michigan School of Medicine Ann Arbor Michigan USA

**Keywords:** drug–drug interactions, mortality, polypharmacy, potentially inappropriate prescribing

## Abstract

**Background:**

Higher‐risk medication use—including regimens with polypharmacy, drug–drug interactions (DDIs), and potentially inappropriate medications (PIMs)—has been linked to adverse outcomes in older adults. However, studies establishing associations between higher‐risk medication use and mortality have lacked measures of organ function and considered types of higher‐risk medication exposures in isolation, allowing potential confounding and overestimation of harm.

**Methods:**

We conducted a prospective cohort study of adults aged ≥ 65 years who took part in the National Health and Nutrition Examination Survey (NHANES) from 1999 to 2016, with mortality follow‐up through 2019. Our primary exposures were three types of higher‐risk medication use: polypharmacy, DDIs, and PIMs. In primary analyses, these were defined, respectively, as follows: being on ≥ 5 prescription medications (polypharmacy); using two medications with ≥ 1 major DDI; and using ≥ 1 prescription PIM. Exposures were determined by medication review during the baseline survey. The primary outcome was all‐cause mortality. Multivariable Cox regression models estimated mortality risk associated with each type of higher‐risk medication use, sequentially adjusting for sociodemographic factors and comorbidities, organ function and health behaviors, and patterns of higher‐risk medication use.

**Results:**

Among 7828 eligible participants (representing 26.5 million adults), 54.3% experienced at least one type of higher‐risk medication use: 40.0% had polypharmacy, 11.4% had a DDI, and 37.6% used a PIM. In fully adjusted models including all higher‐risk medication exposures, only polypharmacy remained significantly associated with increased mortality (hazard ratio [HR] 1.38; 95% CI 1.26–1.51). Sensitivity and subgroup analyses by age and health status yielded generally consistent findings.

**Conclusions:**

When considering multiple types of higher‐risk medication exposure, only polypharmacy was consistently and independently associated with increased mortality among older adults. These results highlight the harms of polypharmacy specifically and could help further target deprescribing interventions to the most high‐risk patients.

## Summary

- Key Points
  ○ In a national sample of older adults, over half had at least one type of higher‐risk medication use: polypharmacy, drug–drug interactions (DDIs), or potentially inappropriate medications (PIMs).
  ○ After adjusting for baseline organ function and overlapping risks, only polypharmacy (use of > 5 medications) was independently linked to increased long‐term mortality.
- Why Does This Paper Matter?
  ○ This study is the first to simultaneously assess associations between multiple types of higher‐risk medication use and mortality.
  ○ The results highlight that polypharmacy is especially and consistently associated with increased long‐term mortality in older Americans, which can reinforce safer prescribing and deprescribing targets.

## Graphical abstract

Having a medication regimen with polypharmacy was associated with higher risk of mortality among older adults even after accounting for clinical factors and other types of higher‐risk medication use.

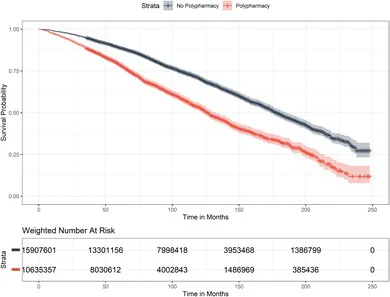

## 1 Introduction

Over 90% of adults ≥ 65 years use at least one medication, and the number with polypharmacy has risen an average of 3% per year over the past two decades [1, 2]. Some of this medication use is undoubtedly appropriate: At a population level, evidence‐based medications are responsible for millions of life‐years saved and up to one‐third of recent gains in life expectancy [3, 4]. However, medications also cause harm. Adverse medication events lead to millions of healthcare visits each year, and older adults are much more likely than younger adults to experience medication‐related harms [5, 6].

There has been growing interest in the concept of deprescribing, or having healthcare professionals carefully reconsider the risk/benefit balance of a medication regimen as a person ages such that medications can be stopped when they are no longer beneficial [7]. Several patterns of higher‐risk medication use have been identified, including: polypharmacy (i.e., using ≥ 5 medications), drug–drug interactions (DDI, i.e., being on medications that together multiply the risk of adverse medication events), and potentially inappropriate medication use (PIM, e.g., certain especially high‐risk medication classes, such as benzodiazepines) [8, 9, 10]. These types of higher‐risk medication use have been considered deprescribing targets given their associations with adverse outcomes, such as all‐cause emergency department visits and hospitalizations [11, 12, 13]. However, prior work characterizing associations between higher‐risk medication use and mortality often does not account in any way for organ function [14]. This is a particularly critical limitation because those who are sicker are likely prescribed more medications, and thus also more likely to have a regimen including a PIM or DDI. Relatedly, most previous work considers each type of higher‐risk medication use in isolation, such as examining polypharmacy (vs. no polypharmacy) but not considering whether a medication regimen includes a DDI. Together, these previous studies are at risk of confounding and may overestimate the harms of each type of higher‐risk medication use.

For many older adults, a higher‐risk medication regimen can be a chronic issue [15]. Clarifying which patterns of higher‐risk medication use are most strongly associated with poor long‐term clinical outcomes can help target deprescribing interventions while ensuring evidence‐based medications are not stopped due to inflated medication regimen harm estimates. We used a nationally representative cohort of community‐dwelling older adults in the United States to explore the independent associations of medication regimens—specifically ones with polypharmacy, DDIs, and PIMs—on mortality risk. We had two a priori hypotheses. First, we hypothesized that the prior literature overstates the association between polypharmacy and mortality by not capturing baseline measures of organ function, a potential confounder of the association between medication burden and mortality. We expected that, after more thoroughly adjusting for baseline organ function and multiple types of higher‐risk medication use than prior studies, DDIs would be the type of medication regimen associated with mortality. Second, given the association between frailty and medication harms, we hypothesized that the oldest adults and those in poorer health, both groups with a higher proportion of frail individuals, would be most susceptible to higher‐risk medication use‐related harms [16].

## 2 Methods

### 2.1 Sample

We included respondents ≥ 65 years who participated in the National Health and Nutrition Examination Survey (NHANES) between 1999 and 2016. NHANES is a cross‐sectional survey that is nationally representative of non‐institutionalized US adults and includes interview, examination, and laboratory components [17]. Respondents not sampled to complete all interview and exam modules or with incomplete data were excluded.

### 2.2 Exposures

Prescription medication use is recorded by NHANES staff during a pill bottle review portion of the interview in which respondents are asked to share the medications they have taken in the prior 30 days. Our primary exposures, all assessed during the same pill bottle review, were three separate indicators of higher‐risk medication use: polypharmacy, DDIs, and PIMs. We defined polypharmacy as using ≥ 5 prescription medications [14, 18]. We defined DDIs using a published list of 10,000 major interactions developed by informaticians who aggregated major interactions from multiple clinical resources [19]; we categorized respondents as having 0 versus ≥ 1 DDI among their prescribed medications. Similar to previous work, we defined PIMs as using at least one of 16 medication classes meeting Beers criteria or with high anticholinergic properties that were associated with high risk of medication‐related harm [20, 21, 22, 23].

To assess the robustness of our results, we included two sensitivity definitions of our exposures. First, we tested our predictors as counts rather than categories (i.e., polypharmacy as the total number of medications in a regimen and DDIs or PIMs as the number of interactions or qualifying medications, respectively). Second, we redefined exposure to a PIM to include only those reported as chronically used (i.e., yes/no used a PIM prescription for at least 60 days prior to the pill bottle review).

### 2.3 Outcome

Death was our primary outcome. We linked each NHANES participant with the Centers for Disease Control and Prevention's public‐use linked mortality files, which allowed us to determine respondent mortality through 2019.

### 2.4 Baseline Characteristics

Using the NHANES survey, we considered four classes of respondent characteristics potentially associated with both medication use and mortality risk: sociodemographic, comorbidity, organ function, and health behaviors. Sociodemographic characteristics included participant age (years), two‐year NHANES cohort entry cycle, sex (male and female), race (non‐Hispanic black, Non‐Hispanic white, Hispanic, and Other), education (less than high school, high school, some college/associates degree, college graduate), and income‐to‐poverty ratio (IPR, continuous). Health status included self‐report history of several diseases (yes, no) including cardiovascular disease (myocardial infarction or stroke), heart failure, chronic obstructive pulmonary disease (COPD), history of liver disease, and a history of non‐skin cancer as well as confirmed anemia (hemoglobin measured at < 11 g/dL). Baseline organ function was operationalized by assessing laboratory and exam measurements of NHANES. Baseline lab values included the presence of elevations in liver enzymes aspartate aminotransferase (AST, > 47 U/L) and alanine aminotransferase (ALT, > 55 U/L), the estimated glomerular filtration rate (mL/min/1.73 m [2]), and glycated hemoglobin units above the cutoff for diabetes (a1c, percentage points above 6.5%); baseline exam elements were systolic and diastolic blood pressure units above guideline recommendations (mmHg above 130 and 90 mmHg, respectively) and body mass index (categorized as normal [≥ 18.5–25 kg/m [2]] vs. abnormal). Health behaviors included smoking status (never smoker, former smoker, and current smoker) and alcohol use (drinks per day or per year, with those having less than one drink per month considered non‐users of alcohol due to NHANES questionnaire skip logic).

Finally, health status is a strong predictor of mortality [24]. We hypothesized that health status would modify the effect of higher‐risk medication use on mortality. We formed this hypothesis based on past work showing an association between frailty and medication harms given that self‐reported health status is itself a predictor of frailty and also strongly correlated with functional limitations [25, 26, 27]. As such, we considered self‐reported general health status (excellent/very good, good, and fair/poor) separately from the other baseline characteristics that we hypothesized to be confounders.

### 2.5 Analysis

We described the cohort and used an Euler diagram to show the proportion with polypharmacy, DDIs, PIM use, or overlapping types of higher‐risk medication use. We then used multivariable logistic regression to separately describe the average marginal effects of participants' baseline characteristics on having each type of higher‐risk medication use. This helps explore whether variables associated with mortality but absent from claims‐based analyses (such as laboratory values) remain associated with higher‐risk medication use patterns after controlling for medical diagnoses, which would help establish the need for this more comprehensive analysis.

We used Cox regression models to assess the association between each type of higher‐risk medication use and mortality. We considered three models. In Model 1, we controlled for the characteristics that might be expected to be available in claims‐based analyses (sociodemographic characteristics and comorbidities) and assessed the impact of each type of higher‐risk medication use on mortality in separate multivariable regressions (i.e., three models to consider the three types of higher‐risk medication use separately). In Model 2, we added the measures of baseline organ function and health behaviors, which are less commonly captured in previous analyses relying on administrative data, and again assessed the association of each type of higher‐risk medication use on mortality in separate multivariable regressions. Finally, in Model 3, we used a single multivariable regression that included all three types of higher‐risk medication use to assess independent associations with mortality.

Finally, we conducted several sensitivity and subgroup analyses using our Model 3. First, we used our alternative coding scheme of our primary exposures (continuous coding and chronically used PIMs, respectively). Second, we limited our follow‐up period to the first 2 years after NHANES participation. Third, we separately stratified the fully adjusted model by (1) self‐reported general health status (excellent/very good, good, and fair/poor) and by (2) age (65–74, ≥ 75 years). Fourth, we limited our analysis to individuals who reported a diagnosis of cardiovascular disease to further assess the robustness of our results among a more homogenous population more likely to qualify for similar medications. Analyses were performed in R version 4.4.1 using the *survey* package using two‐tailed tests and an alpha = 0.05. NHANES intentionally oversamples certain groups to be able to generate nationally representative statistics, and this includes underrepresented groups that have higher rates of mortality. In our analyses, counts are unweighted unless otherwise specified, and all other estimates are weighted to account for nonresponse and sampling to be representative to the non‐institutionalized population.

## 3 Results

Of 12,551 adults aged ≥ 65 years, 7828 participants were sampled to complete the survey, laboratory, and exam components and had complete data. This analytic sample was similar to the overall NHANES sample (Table S1) and represented a weighted population of 26.5 million adults ≥ 65 years. Participants were followed for a mean 8.5 years during which time there were 3591 (representing 10.7 million deaths when weighted, see Table S2 for mortality by survey cycle). The sample median age was 72 years (interquartile range [IQR] 68–78), 55.0% were female (95% CI 53.9%–56.1%), and 26.7% were college graduates (21.7%–25.6%, Table 1). Patients were using a median 4 medications (IQR 2–6).

**TABLE 1:** Characteristics of analytic cohort.

Category	Variable		Overall (*n* = 7828)
Demographics	Cohort entry, % (95% CI)	1999–2004	26.0% (23.4–28.5)
		2005–2010	33.3% (30.3–36.4)
		2011–2016	40.7% (37.6–43.9)
	Age, median (IQR), years		72 (68–78)
	Female, % (95% CI)		57.4% (56.1–58.7)
	Education, % (95% CI)	College graduate	23.7% (21.7–25.6)
		Some college	26.8% (25.3–28.4)
		High school	25.8% (24.4–27.2)
		Less than high school	23.7% (21.7–25.8)
	Race/Ethnicity, % (95% CI)	Non‐Hispanic white	82.9% (81.1–84.8)
		Hispanic	6.3% (5.0–7.6)
		Non‐Hispanic black	6.9% (6.0–7.9)
		Other	3.9% (3.2–5.6)
	Income‐to‐poverty ratio, median (IQR)		2.6 (1.6–4.4)
Comorbidities	Cardiovascular disease, % (95% CI)	Yes	16.9% (15.8–18.0)
	Anemia, % (95% CI)	Yes	1.8% (1.4%–2.1%)
	Congestive heart failure, % (95% CI)	Yes	8.0% (7.1–8.8)
	Chronic obstructive pulmonary disease, % (95% CI)	Yes	11.9% (10.8–13.0)
	Liver disease, % (95% CI)	Yes	3.4% (2.9%–3.9%)
	History of cancer, % (95% CI)	Yes	27.1% (25.8–28.4)
Organ function	AST, U/L median (IQR)		24 (20–28)
	ALT, U/L median (IQR)		20 (16–25)
	Systolic blood pressure, mmHg median (IQR)		134 (122–148)
	Diastolic blood pressure, mmHg median (IQR)		68 (60–76)
	BMI, kg/m^2^ median (IQR)		27.6 (24.6–31.4)
	A1c, % median (IQR)		5.7 (5.4–6.0)
	eGFR, mL/min/1.73m^2^ median (IQR)		75.3 (61.3–89.3)
Health Behaviors	Smoking status, % (95% CI)	Never smoker	48.2% (46.7–49.7)
		Current smoker	9.0% (8.2–9.8)
		Former smoker	42.8% (41.3–44.3)
	Alcoholic drinks per year, median (IQR)		0 (0–104)

Abbreviations: a1c, glycated hemoglobin as a percentage; ALT, alanine aminotransferase; AST, aspartate aminotransferase; BMI, body mass index in kg/m^2^; eGFR, estimated glomerular filtration rate in mL/min/1.73 m^2^.

**FIGURE 1:**
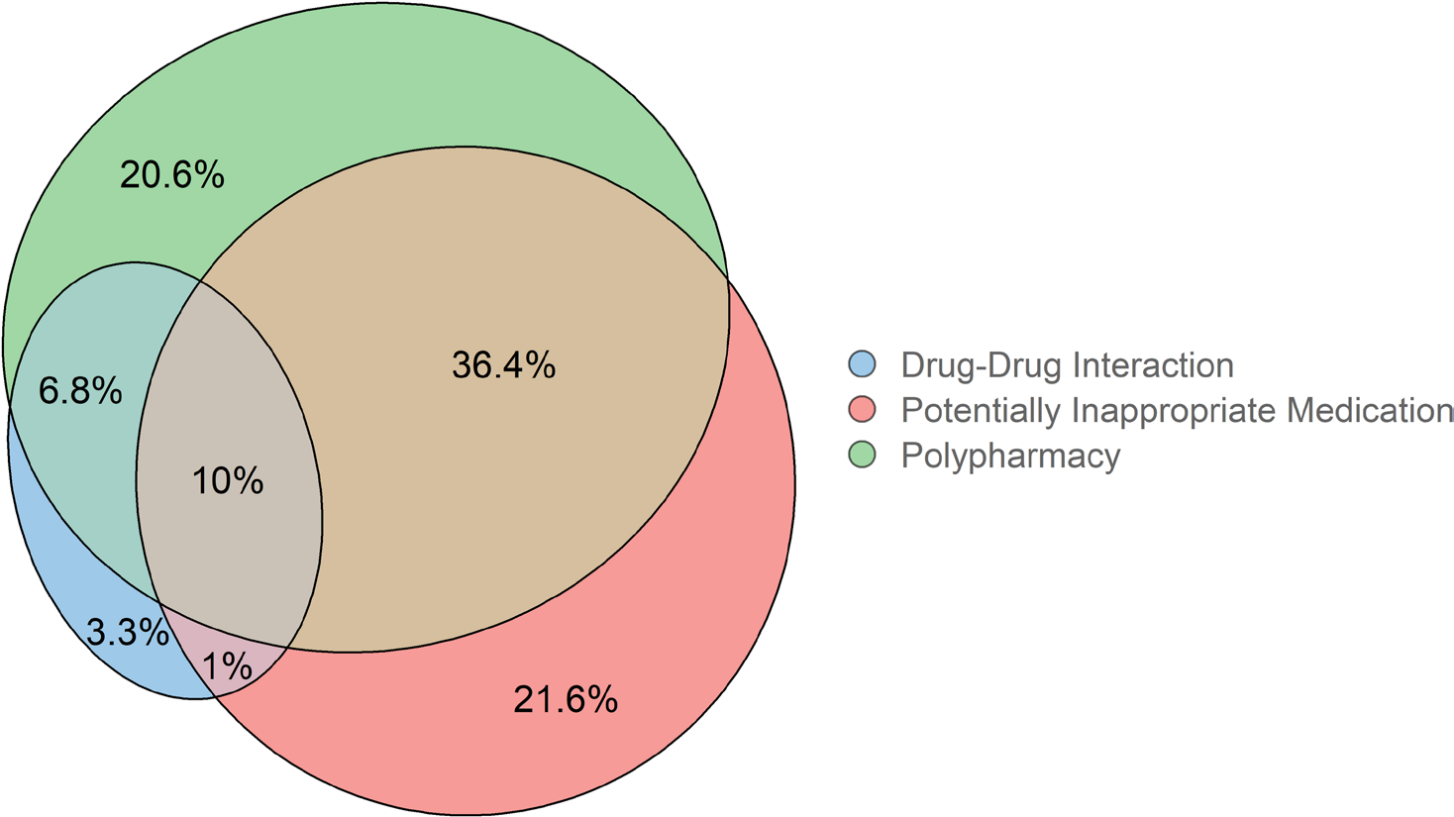
Overlapping higher‐risk medications use.

The majority (54.3%) of participants had at least one type of higher‐risk medication use. 40.0% (95% CI 38.5%–41.6%), 11.4% (95% CI 10.5%–12.5%), and 37.6% (95% CI 36.0%–39.1%) had polypharmacy, a DDI, or a PIM, respectively. Of those with polypharmacy, the median number of medications used was 7 (IQR 5–9), and 7.3% were using ≥ 10 medications (95% CI 6.6%–8.1%). Of those with a DDI among their regimen, the median had 2 unique interactions (IQR 2–4). Of those with a PIM in their regimen, the median was using one such medication (IQR 1–2). Of those with at least one type of higher‐risk medications use, the largest proportion (36.4%, 95% CI 34.2%–38.6%) had both polypharmacy and were using a PIM (Figure 1). The fewest participants had both a DDI and PIM use (1.0%, 95% CI 0.6%–1.4%). In fully adjusted models, multiple aspects of baseline organ function were associated with all three types of higher‐risk medication use (Table S3).

**FIGURE 2:**
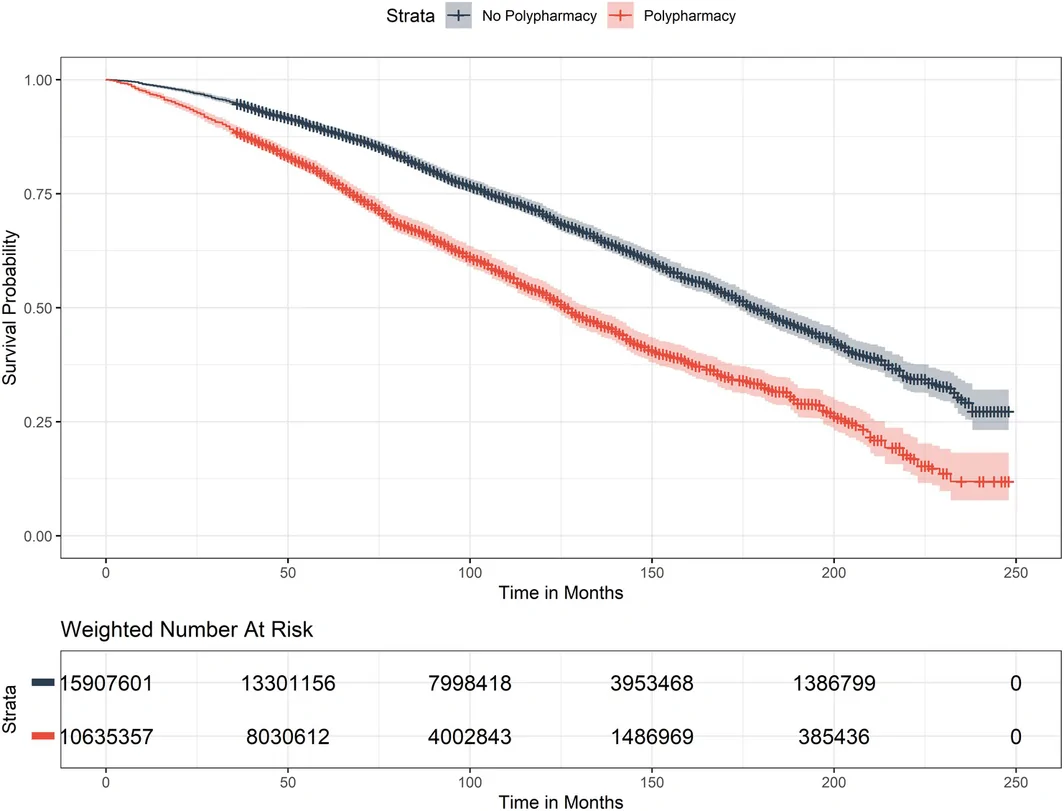
Kaplan–Meier curves showing higher‐risk medication use and mortality Model 3: Hazard ratio 1.38, 95% CI 1.26–1.51. Abbreviations: CI, Confidence Interval.

In the survival analyses, the three types of higher‐risk medication use were each independently associated with an increased risk of mortality in Model 1 (adjusting for sociodemographic characteristics and comorbidities) and Model 2 (Model 1 additionally adjusting for baseline organ function and health behaviors). However, in Model 3, which included all three types of higher‐risk medication use, only polypharmacy remained associated with mortality (hazard ratio [HR] 1.38, 95% CI 1.26–1.51; Figure 2), while PIM and DDI were not significantly associated with mortality (Table 2 and Figure S1). Of note, in this model, the variance inflation factor for polypharmacy, PIM, and DDI was < 3.

**TABLE 2:** Association between higher‐risk medication use and mortality.

	Model 1		Model 2		Model 3	
	HR (95% CI)	*p*	HR (95% CI)	*p*	HR (95% CI)	*p*
Polypharmacy	1.48 (1.37–1.61)	< 0.01	1.41 (1.31–1.53)	< 0.01	1.38 (1.26–1.51)	< 0.01
Potentially inappropriate medication	1.21 (1.13–1.30)	< 0.01	1.18 (1.10–1.27)	< 0.01	1.05 (0.97–1.14)	0.24
Drug–drug interaction	1.16 (1.03–1.31)	0.02	1.14 (1.02–1.29)	0.02	1.03 (0.91–1.16)	0.67

*Note:* Model 1: Each row is a separate regression adjusted for sociodemographic characteristics and comorbidities. Model 2: Each row is a separate regression additionally adjusted for disease control. Model 3: One regression additionally adjusted for all types of higher‐risk medication use.

Abbreviation: HR, Hazard Ratio.

In sensitivity analysis using continuous coding for each exposure, only number of medications (HR 1.07, 95% CI 1.05–1.09)—the polypharmacy outcome in continuous form—was significantly associated with higher mortality. Increasing numbers of PIM were associated with lower hazard of mortality (HR 0.94, 95% CI 0.89–0.99), and the number of DDIs (HR 1.00, 95% CI 0.97–1.03) was not associated with mortality. When PIM exposure required self‐reported chronic use of the medication, there remained no association with mortality in the fully adjusted model (HR 1.01, 95% CI 0.93–1.09) while polypharmacy estimates remained unchanged (HR 1.40, 95% CI 1.28–1.54). In sensitivity analyses limited to the first 2 years of follow‐up, results were similar to the main analysis (Figures S2–S4).

In stratified analysis, when limiting the sample to participants ≥ 75 years (*n* = 3437, followed for an average 7.2 years), only polypharmacy (HR 1.31, 95% CI 1.16–1.49) was associated with mortality. Similarly, in those age 65–74 years (*n* = 4391, followed for an average 9.3 years), again only polypharmacy was associated with mortality (HR 1.39, 95% CI 1.18–1.64, Table 3). When stratifying the primary analysis by general health status, only polypharmacy was associated with mortality among those with good health (HR 1.35, 95% CI 1.16–1.58) and fair to poor health (HR 1.35, 95% CI 1.14–1.60). The association between polypharmacy and mortality was not statistically significant at the prespecified alpha value among those excellent to very good health (HR 1.20 95% CI 0.99–1.45), though the confidence interval spanned potentially clinically significant values. When the sample was limited to individuals with cardiovascular disease (*n* = 1375), polypharmacy (and not DDI or PIM) remained associated with mortality (HR 1.36, 95% CI 1.23–1.51).

**TABLE 3:** Fully adjusted survival analysis stratified by health status or age.

	65–74 (*n* = 4391)		≥ 75 (*n* = 3437)		Excellent/Very good (*n* = 2801)		Good (*n* = 2774)		Fair/Poor (*n* = 2245)	
	HR (95% CI)	*p*	HR (95% CI)	*p*	HR (95% CI)	*p*	HR (95% CI)	*p*	HR (95% CI)	*p*
Polypharmacy	1.39 (1.18–1.64)	< 0.01	1.31 (1.16–1.49)	< 0.01	1.20 (0.99–1.45)	0.06	1.35 (1.15–1.59)	< 0.01	1.34 (1.13–1.59)	< 0.01
Potentially inappropriate medication	1.09 (0.95–1.27)	0.22	1.03 (0.94–1.14)	0.51	1.11 (0.94–1.32)	0.22	0.99 (0.84–1.17)	0.94	1.00 (0.88–1.14)	0.99
Drug–drug interaction	1.09 (0.89–1.33)	0.40	1.00 (0.85–1.18)	0.97	0.98 (0.76–1.25)	0.84	0.85 (0.70–1.05)	0.13	1.16 (0.97–1.40)	0.11

*Note:* Death during following up: Age 65–74 1,399, age > 75 2,192, excellent/very good health 1117, good 1229, fair/poor 1242. 8 patients from analytic samples used for primary analysis excluded due to missing self‐reported general health status in NHANES.

Abbreviation: HR, Hazard Ratio.

## 4 Discussion

In a national sample of community‐dwelling older adults, polypharmacy was consistently associated with higher mortality, even after adjusting for objective measures of baseline organ function and other types of higher‐risk medication use. While DDIs and PIMs were both associated with increased mortality risk when considered separately, these associations weakened to the point of statistical insignificance when also considering polypharmacy. The only association between higher‐risk medication use and lower mortality, which was between PIMs and mortality, was small and not consistent when varying the exposure definition. These findings suggest that while clinicians should remain vigilant regarding specific medication quality, the total number of medications may be the important clinical data point for identifying older adults at the highest risk for adverse outcomes.

Even after adjusting for the presence or absence of disease (as would be possible in claims datasets), measures of baseline organ function remained associated with polypharmacy. The existing literature has described how certain demographic characteristics may be associated with higher‐risk medication regimens, and we add to it by showing how measures of organ function, not just multimorbidity operationalized as a binary or count variable, are also associated with higher‐risk medication use [28, 29, 30]. As such, studies that do not control for measures of organ function may be biased toward finding associations between potentially harmful patterns of medication use and mortality. This highlights the limitations of using purely administrative data to study the impacts of higher‐risk medication use.

However, our main hypothesis that polypharmacy would not remain a significant predictor of mortality when accounting for clinical measures and other types of higher‐risk medication use was incorrect. Many older adults experience unwanted effects of medications [31, 32]. While these adverse effects range in severity, the high absolute rates of medication use among older adults with polypharmacy could mean that even though serious adverse events related to mortality are relatively rare for an individual (e.g., fall complicated by hip fracture), they will occur at meaningful rates at the population level. There is also bioplausibility linking polypharmacy use with longer term outcomes. The persistence of the association between baseline polypharmacy and long‐term mortality could suggest a potential legacy effect of high medication burden. Preclinical studies in aging rat models and other cohort studies have demonstrated that chronic exposure to multiple medications can result in impaired physical and cognitive function independent of underlying disease in the long‐term [33, 34, 35]. The hypothesis that polypharmacy may act as a chronic physiologic stressor, depleting homeostatic reserves over time, as seen in animal models and retrospective epidemiological work, requires additional study to further elucidate mechanisms in prospective cohorts. This may also explain why DDIs and PIMs were not consistently associated with mortality. Whereas polypharmacy can contribute to mortality risk via cumulative metabolic and physiological strain, these other patterns of higher‐risk medication use act on mortality risk only through more direct mechanisms (e.g., causing an arrhythmia), which could require significantly more powerful studies to detect. Notably, because it is plausible that DDIs and PIMs are on the causal pathway linking polypharmacy and mortality, our results could be biased towards the null in our fully adjusted model. However, the relative stability of the effect size of polypharmacy across models suggests that the medication count itself exerts a risk to survival that is independent of the specific appropriateness or interaction profile of the drugs, perhaps reflecting the cumulative physiological stress of multi‐drug metabolism. Residual confounding may remain, as we do not have access to all possible comorbidities or measures of organ function. However, this is one of the most comprehensive assessments of the association between medication use and mortality in the literature to date, and the consistency of our results across subgroups and sensitivity analyses suggests confounding does not fully account for our findings.

We also hypothesized that higher‐risk medication use would only be associated with mortality in the oldest adults and those in poorer health—groups that would have a higher proportion of frail individuals and therefore be more likely to experience adverse outcomes. Unexpectedly, polypharmacy remained a significant predictor of mortality at most levels of general health status and across age groups. It is therefore important to consider higher‐risk medication use in all older adults, not just those that one would consider at higher baseline risk for poor outcomes. However, while self‐reported poor health status is associated with being classified as frail, the correlation is not perfect [26]. As such, there is still a need to further understand which populations are at highest risk of adverse events from higher‐risk medication use.

This study adds to the existing literature in three notable ways. First, we include measures of organ function. Most of the largest studies exploring the association between higher‐risk medication use and mortality rely on insurance claims, which lack clinical and laboratory measures that could confound the association between medication use and mortality [13, 14, 36]. Second, to our knowledge, this is the first study that accounts for the independent associations of three types of higher‐risk medication use on mortality. This is in contrast with earlier studies that have used NHANES, which tend also to be limited to individuals with specific diseases (such as chronic kidney disease) and not include such a breadth of clinical measures [37]. Finally, many prior cohort studies were conducted outside the United States [38, 39]. Because different medications are approved and available in different countries, and because higher‐risk medication use differs by geographic region, this could represent a threat to the external validity of previous work [40].

These results may be useful when considering inclusion criteria for trials of medication de‐escalation. For example, the Optimizing Treatment for Mild Systolic Hypertension in the Elderly (OPTIMIZE) study randomized older adults with blood pressures < 150/90 mmHg on multiple antihypertensives to either have their antihypertensive regimen de‐escalated or continued [41]. While polypharmacy was one of several potential inclusion criteria that could be used for enrollment into the trial, most patients enrolled were using fewer than 5 medications, suggesting those at the highest risk of medication harms may not have been captured. Results from the trial suggested de‐escalation would not be cost‐effective and would not reduce hospitalizations, but it is unknown if findings would have been different had the trial been enriched for higher risk groups, such as those with polypharmacy [42, 43]. Our results suggest that a focused approach to deprescribing initiatives that explicitly focuses on medication classes of interest (e.g., antihypertensives) specifically among those with polypharmacy might maximize likelihood of benefit and ensure efficient trials enriched with the highest risk populations.

This study has several limitations. First, we include only community‐dwelling older adults. While this represents over 93% of older adults, the results may not generalize to older adults living in residential settings [44]. Specifically, those adults living in residential settings may be less likely to benefit from medications for chronic diseases and more likely to be harmed by them [45]. Second, despite our efforts, we cannot fully capture patient clinical complexity. Some of these complexities include drug‐organ interactions (e.g., being on a renally cleared medication when having chronic kidney disease), which should be assessed in future studies. Relatedly, there may still be residual confounding from diseases or laboratory measures (e.g., B‐type natriuretic peptide as a measure of heart failure severity) that we could not assess because they are not reliably included in NHANES. Third, while NHANES medication review capture medications recently taken (rather than simply prescribed) and is regularly used to describe trends in polypharmacy among older adults, the 30‐day reporting period makes it possible that not all reviewed medications were being used concurrently [29, 30]. Fourth, we identified patients at the time of cohort entry and conducted our intention‐to‐treat analysis based on the prescribed medications used at that timepoint. This means we cannot account for time‐varying factors, such as changes in health status or medication adherence that occurred after the baseline period. The alignment of the outcomes in the primary analysis and the sensitivity analysis restricted to the first 2 years after NHANES enrollment is reassuring, and there is evidence that polypharmacy is a relatively chronic state for many older adults, but our results should be interpreted within the limitation of not accounting for time‐varying confounders [15]. Fifth, our analysis stopped before the COVID pandemic because this is the most recent linked data available. Sixth, these findings are not causal; they do not suggest reducing higher‐risk medication use will necessarily improve outcomes, and the benefits of many medication regimens with polypharmacy, DDIs, and PIMs may outweigh the harms. Finally, numerous other potential harms from medication use, such as falls, hospitalizations, and patient quality of life, are likely more common and even potentially more addressable than mortality. The design of NHANES precluded our ability to study how higher‐risk medication use affects these important outcomes, which should be the focus of future studies.

Limitations notwithstanding, we find consistent evidence of an association between medication regimens with polypharmacy, but not PIMs or DDIs, and death. These estimates more completely characterize the association between certain patterns of medication use and mortality, help clarify how an older adult's pattern of medication use correlates with their future mortality risk, and may provide guidance about which types of patients would be ideal targets for trials studying medication de‐escalation.

## Author Contributions

Conceptualization: Chaitoff, Maust. Data gathering and analysis: Chaitoff. Interpretation of data: Chaitoff, Jungo, Min, Maust. Drafting of initial draft: Chaitoff. Critical review of the manuscript: Chaitoff, Jungo, Min, Maust. Supervision: Maust.

## Disclosure

Support was also provided by the Department of Veterans Affairs, Veterans Health Administration, Health Systems Research Service. The views expressed in this article are those of the authors and do not necessarily reflect the position or policy of the Department of Veterans Affairs or the United States government. Dr. Chaitoff reports serving as a consultant for Alosa Health and AristaMD. This is unrelated to the current project. Dr. Maust is supported by NIH grant R01AG074957. Dr. Jungo is supported by a Postdoc. Mobility Fellowship from the Swiss National Science Foundation [P500PM_206728]. This work had no direct sponsor.

## Conflicts of Interest

Dr. Chaitoff reports serving as a consultant for Alosa Health and AristaMD. This is unrelated to the current project.

## Supporting information


**Figure S1:** Adjusted hazard ratios from fully adjusted Model 3.
**Table S1:** Weighted proportion of total NHANES sample aged > 65 years and those with complete data included in the analytic cohort.
**Table S2:** Proportion of respondents experiencing death by cohort entry cycle.
**Table S3:** Average marginal effects on probability of medication regimen with polypharmacy, potentially inappropriate medications use, or a drug–drug interaction.
**Figure S2:** Event‐free survival by polypharmacy status by follow‐up time.
**Figure S3:** Event‐free survival by potentially inappropriate medication use status by follow‐up time.
**Figure S4:** Event‐free survival by drug–drug interaction status by follow‐up time.

## References

[1] G. K. Innes , C. L. Ogden , V. Crentsil , J. Concato , and T. H. Fakhouri , “Prescription Medication Use Among Older Adults in the US,” JAMA Internal Medicine 184, no. 9 (2024): 1121–1123, 10.1001/jamainternmed.2024.2781. 38949837 PMC11217884

[2] X. Wang , K. Liu , K. Shirai , et al., “Prevalence and Trends of Polypharmacy in U.S. Adults, 1999–2018,” Global Health Research and Policy 8 (2023): 25, 10.1186/s41256-023-00311-4. 37434230 PMC10337167

[3] J. D. Buxbaum , M. E. Chernew , A. M. Fendrick , and D. M. Cutler , “Contributions of Public Health, Pharmaceuticals, and Other Medical Care to US Life Expectancy Changes, 1990‐2015,” Health Affairs (Project Hope) 39, no. 9 (2020): 1546–1556, 10.1377/hlthaff.2020.00284. 32897792

[4] F. Lichtenberg , “How Many Life‐Years Have New Drugs Saved? A 3‐Way Fixed‐Effects Analysis of 66 Diseases in 27 Countries, 2000‐2013,” International Healh 11 (2019): 403–416, 10.3386/w25483. 30912800

[5] U. Sarkar , A. López , J. H. Maselli , and R. Gonzales , “Adverse Drug Events in U.S. Adult Ambulatory Medical Care,” Health Services Research 46, no. 5 (2011): 1517–1533, 10.1111/j.1475-6773.2011.01269.x. 21554271 PMC3168717

[6] D. S. Budnitz , D. A. Pollock , K. N. Weidenbach , A. B. Mendelsohn , T. J. Schroeder , and J. L. Annest , “National Surveillance of Emergency Department Visits for Outpatient Adverse Drug Events,” Journal of the American Medical Association 296, no. 15 (2006): 1858–1866, 10.1001/jama.296.15.1858. 17047216

[7] E. Reeve , D. Gnjidic , J. Long , and S. Hilmer , “A Systematic Review of the Emerging Definition of ‘Deprescribing’ With Network Analysis: Implications for Future Research and Clinical Practice,” British Journal of Clinical Pharmacology 80, no. 6 (2015): 1254–1268, 10.1111/bcp.12732. 27006985 PMC4693477

[8] A. M. Linsky , A. Motala , M. Booth , E. Lawson , and P. G. Shekelle , “Deprescribing in Community‐Dwelling Older Adults: A Systematic Review and Meta‐Analysis,” JAMA Network Open 8, no. 5 (2025): e259375, 10.1001/jamanetworkopen.2025.9375. 40338546 PMC12062908

[9] S. Williams , G. Miller , R. Khoury , and G. T. Grossberg , “Rational Deprescribing in the Elderly,” Annals of Clinical Psychiatry: Official Journal of the American Academy of Clinical Psychiatrists 31, no. 2 (2019): 144–152. 31046036

[10] E. E. Vasilevskis , A. S. Shah , E. K. Hollingsworth , et al., “Deprescribing Medications Among Older Adults From End of Hospitalization Through Postacute Care: A Shed‐MEDS Randomized Clinical Trial,” JAMA Internal Medicine 183, no. 3 (2023): 223–231, 10.1001/jamainternmed.2022.6545. 36745422 PMC9989899

[11] L. Létinier , J. Bezin , A. Jarne , and A. Pariente , “Drug‐Drug Interactions and the Risk of Emergency Hospitalizations: A Nationwide Population‐Based Study,” Drug Safety 46, no. 5 (2023): 449–456, 10.1007/s40264-023-01283-7. 37046156

[12] S. J. Davies , D. Rudoler , C. de Oliveira , A. Huang , P. Kurdyak , and A. Iaboni , “Comparative Safety of Chronic Versus Intermittent Benzodiazepine Prescribing in Older Adults: A Population‐Based Cohort Study,” Journal of Psychopharmacology (Oxford, England) 36, no. 4 (2022): 460–469, 10.1177/02698811211069096. 35102786 PMC9066681

[13] J. Chae , H. J. Cho , S. H. Yoon , and D. S. Kim , “The Association Between Continuous Polypharmacy and Hospitalization, Emergency Department Visits, and Death in Older Adults: A Nationwide Large Cohort Study,” Frontiers in Pharmacology 15 (2024): 1382990, 10.3389/fphar.2024.1382990. 39144630 PMC11322047

[14] T. I. Chang , H. Park , D. W. Kim , et al., “Polypharmacy, Hospitalization, and Mortality Risk: A Nationwide Cohort Study,” Scientific Reports 10 (2020): 18964, 10.1038/s41598-020-75888-8. 33144598 PMC7609640

[15] J. W. Wastesson , L. Morin , M. L. Laroche , and K. Johnell , “How Chronic Is Polypharmacy in Old Age? A Longitudinal Nationwide Cohort Study,” Journal of the American Geriatrics Society 67, no. 3 (2019): 455–462, 10.1111/jgs.15717. 30575952

[16] L. Ye , D. Nieboer , J. Yang‐Huang , et al., “The Association Between Frailty and the Risk of Medication‐Related Problems Among Community‐Dwelling Older Adults in Europe,” Journal of the American Geriatrics Society 71, no. 8 (2023): 2485–2494, 10.1111/jgs.18343. 36965170

[17] CDC , “About NHANES. National Health and Nutrition Examination Survey,” https://www.cdc.gov/nchs/nhanes/about/index.html.

[18] N. Masnoon , S. Shakib , L. Kalisch‐Ellett , and G. E. Caughey , “What Is Polypharmacy? A Systematic Review of Definitions,” BMC Geriatrics 17, no. 1 (2017): 230, 10.1186/s12877-017-0621-2. 29017448 PMC5635569

[19] E. Kontsioti , S. Maskell , B. Dutta , and M. Pirmohamed , “A Reference Set of Clinically Relevant Adverse Drug‐Drug Interactions,” Scientific Data 9, no. 1 (2022): 72. 35246559 10.1038/s41597-022-01159-y PMC8897500

[20] K. T. Jungo , N. K. Choudhry , A. Chaitoff , and J. C. Lauffenburger , “Associations Between Sex, Race/Ethnicity, and Age and the Initiation of Chronic High‐Risk Medication in US Older Adults,” Journal of the American Geriatrics Society 72 (2024): 3705–3718, 10.1111/jgs.19173. 39215549 PMC11637294

[21] By the 2019 American Geriatrics Society Beers Criteria Update Expert Panel , “American Geriatrics Society 2019 Updated AGS Beers Criteria for Potentially Inappropriate Medication Use in Older Adults,” Journal of the American Geriatrics Society 67, no. 4 (2019): 674–694, 10.1111/jgs.15767. 30693946

[22] J. L. Rudolph , M. J. Salow , M. C. Angelini , and R. E. McGlinchey , “The Anticholinergic Risk Scale and Anticholinergic Adverse Effects in Older Persons,” Archives of Internal Medicine 168, no. 5 (2008): 508–513, 10.1001/archinternmed.2007.106. 18332297

[23] K. T. Jungo , N. K. Choudhry , and J. C. Lauffenburger , “Sociodemographic Differences in Discontinuation of High‐Risk Medications: A Retrospective Cohort Study,” NPJ Aging 12, no. 1 (2025): 13, 10.1038/s41514-025-00310-4. 41413378 PMC12820038

[24] G. Lorem , S. Cook , D. A. Leon , N. Emaus , and H. Schirmer , “Self‐Reported Health as a Predictor of Mortality: A Cohort Study of Its Relation to Other Health Measurements and Observation Time,” Scientific Reports 10, no. 1 (2020): 4886, 10.1038/s41598-020-61603-0. 32184429 PMC7078209

[25] S. Cullinan , D. O'Mahony , D. O'Sullivan , and S. Byrne , “Use of a Frailty Index to Identify Potentially Inappropriate Prescribing and Adverse Drug Reaction Risks in Older Patients,” Age and Ageing 45, no. 1 (2016): 115–120, 10.1093/ageing/afv166. 26683048

[26] A. M. González‐Pichardo , A. P. Navarrete‐Reyes , H. Adame‐Encarnación , et al., “Association Between Self‐Reported Health Status and Frailty in Community‐Dwelling Elderly,” Journal of Frailty & Aging 3, no. 2 (2014): 104–108, 10.14283/jfa.2014.9. 27049902

[27] E. L. Idler , L. B. Russell , and D. Davis , “Survival, Functional Limitations, and Self‐Rated Health in the NHANES I Epidemiologic Follow‐Up Study, 1992. First National Health and Nutrition Examination Survey,” American Journal of Epidemiology 152, no. 9 (2000): 874–883, 10.1093/aje/152.9.874. 11085400

[28] A. Rieckert , U. S. Trampisch , R. Klaaßen‐Mielke , et al., “Polypharmacy in Older Patients With Chronic Diseases: A Cross‐Sectional Analysis of Factors Associated With Excessive Polypharmacy,” BMC Family Practice 19, no. 1 (2018): 113, 10.1186/s12875-018-0795-5. 30021528 PMC6052592

[29] E. D. Kantor , C. D. Rehm , J. S. Haas , A. T. Chan , and E. L. Giovannucci , “Trends in Prescription Drug Use Among Adults in the United States From 1999‐2012,” Journal of the American Medical Association 314, no. 17 (2015): 1818–1831, 10.1001/jama.2015.13766. 26529160 PMC4752169

[30] C. J. Charlesworth , E. Smit , D. S. H. Lee , F. Alramadhan , and M. C. Odden , “Polypharmacy Among Adults Aged 65 Years and Older in the United States: 1988‐2010,” Journals of Gerontology. Series A, Biological Sciences and Medical Sciences 70, no. 8 (2015): 989–995, 10.1093/gerona/glv013. 25733718 PMC4573668

[31] E. A. Chrischilles , E. T. Segar , and R. B. Wallace , “Self‐Reported Adverse Drug Reactions and Related Resource Use. A Study of Community‐Dwelling Persons 65 Years of Age and Older,” Annals of Internal Medicine 117, no. 8 (1992): 634–640, 10.7326/0003-4819-117-8-634. 1530194

[32] E. A. Chrischilles , R. VanGilder , K. Wright , M. Kelly , and R. B. Wallace , “Inappropriate Medication Use as a Risk Factor for Self‐Reported Adverse Drug Effects in Older Adults,” Journal of the American Geriatrics Society 57, no. 6 (2009): 1000–1006, 10.1111/j.1532-5415.2009.02269.x. 19507293

[33] A. Huizer‐Pajkos , A. E. Kane , S. E. Howlett , et al., “Adverse Geriatric Outcomes Secondary to Polypharmacy in a Mouse Model: The Influence of Aging,” Journals of Gerontology. Series A, Biological Sciences and Medical Sciences 71, no. 5 (2016): 571–577, 10.1093/gerona/glv046. 25940962 PMC5007735

[34] S. N. Hilmer , D. E. Mager , E. M. Simonsick , et al., “A Drug Burden Index to Define the Functional Burden of Medications in Older People,” Archives of Internal Medicine 167, no. 8 (2007): 781–787, 10.1001/archinte.167.8.781. 17452540

[35] D. Gnjidic , S. N. Hilmer , F. M. Blyth , et al., “Polypharmacy Cutoff and Outcomes: Five or More Medicines Were Used to Identify Community‐Dwelling Older Men at Risk of Different Adverse Outcomes,” Journal of Clinical Epidemiology 65, no. 9 (2012): 989–995, 10.1016/j.jclinepi.2012.02.018. 22742913

[36] S. Perreault , M. E. Schnitzer , E. Disso , J. Qazi , L. A. Boivin‐Proulx , and M. Dorais , “Polypharmacy and Risk of Mortality Among Patients With Heart Failure Following Hospitalization: A Nested Case–Control Study,” Scientific Reports 12, no. 1 (2022): 19963, 10.1038/s41598-022-24285-4. 36402903 PMC9675839

[37] X. Wang , C. Yang , J. Jiang , Y. Hu , Y. Hao , and J. Y. Dong , “Polypharmacy, Chronic Kidney Disease, and Mortality Among Older Adults: A Prospective Study of National Health and Nutrition Examination Survey, 1999–2018,” Frontiers in Public Health 11 (2023): 1116583, 10.3389/fpubh.2023.1116583. 37033012 PMC10077868

[38] Y. T. Huang , A. Steptoe , L. Wei , and P. Zaninotto , “The Impact of High‐Risk Medications on Mortality Risk Among Older Adults With Polypharmacy: Evidence From the English Longitudinal Study of Ageing,” BMC Medicine 19, no. 1 (2021): 321, 10.1186/s12916-021-02192-1. 34911547 PMC8675465

[39] L. E. Davies , A. Kingston , A. Todd , and B. Hanratty , “Is Polypharmacy Associated With Mortality in the Very Old: Findings From the Newcastle 85+ Study,” British Journal of Clinical Pharmacology 88, no. 6 (2022): 2988–2995, 10.1111/bcp.15211. 34981552 PMC9302636

[40] F. Tian , Z. Chen , Y. Zeng , Q. Feng , and X. Chen , “Prevalence of Use of Potentially Inappropriate Medications Among Older Adults Worldwide: A Systematic Review and Meta‐Analysis,” JAMA Network Open 6, no. 8 (2023): e2326910, 10.1001/jamanetworkopen.2023.26910. 37531105 PMC10398411

[41] J. P. Sheppard , J. Burt , M. Lown , et al., “Effect of Antihypertensive Medication Reduction vs Usual Care on Short‐Term Blood Pressure Control in Patients With Hypertension Aged 80 Years and Older: The OPTIMISE Randomized Clinical Trial,” Journal of the American Medical Association 323, no. 20 (2020): 2039–2051, 10.1001/jama.2020.4871. 32453368 PMC7251449

[42] J. P. Sheppard , E. Temple , A. Wang , et al., “Effect of Antihypertensive Deprescribing on Hospitalisation and Mortality: Long‐Term Follow‐Up of the OPTiMISE Randomised Controlled Trial,” Lancet Healthy Longevity 5, no. 8 (2024): e563–e573, 10.1016/S2666-7568(24)00131-4. 39094592 PMC11327766

[43] S. Jowett , S. Kodabuckus , G. A. Ford , et al., “Cost‐Effectiveness of Antihypertensive Deprescribing in Primary Care: A Markov Modelling Study Using Data From the OPTiMISE Trial,” Hypertension 79, no. 5 (2022): 1122–1131, 10.1161/HYPERTENSIONAHA.121.18726. 35266409 PMC8997697

[44] L. Pillsbury , E. A. Miller , C. Boon , et al., “Size and Demographics of Aging Populations,” in Providing Healthy and Safe Foods as We Age: Workshop Summary (National Academies Press (US), 2010). 21391340

[45] A. Benetos , S. Gautier , A. Freminet , et al., “Reduction of Antihypertensive Treatment in Nursing Home Residents,” New England Journal of Medicine 393 (2025): 1990–2000, 10.1056/NEJMoa2508157. 40879421

