# Supplementary material for: Assessing Associations of Three Types of Higher‐Risk Medication Use and Mortality in Older Adults
Source: J Am Geriatr Soc. 2026 Jul 8;74(8):2308–16. doi: 10.1111/jgs.70569 (PMC13496343; doi:10.1111/jgs.70569)
Supplement: Supplementary file 1 [file JGS-74-2308-s001.pdf]

Supplemental Figure 1. Adjusted hazard ratios from fully adjusted model 3

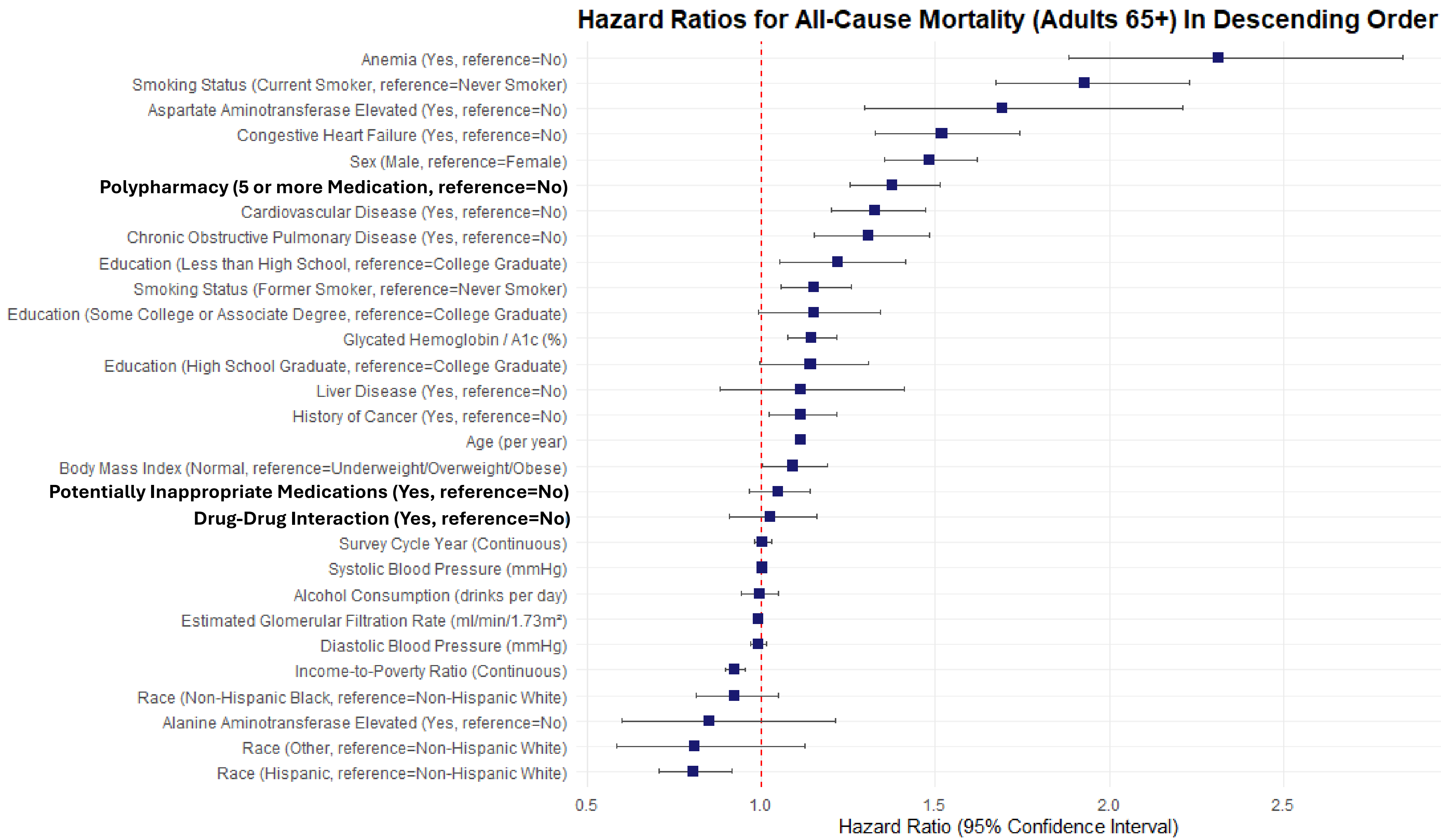

Supplemental Table 1. Weighted proportion of total NHANES sample aged ≥ 65 years and those with complete data included in the analytic cohort

**Variable**	**All Adults ≥ 65 Years**	**Adults ≥ 65 Years in Analytic Cohort**
**Age, mean years**	73.5	73.1
**Sex, % female**	43.5	45.0
**Race, %**		
Non-Hispanic White	80.4	82.9
Non-Hispanic Black	8.1	6.9
Hispanic, Any Race	6.8	6.3
Non-Hispanic, Other Race	4.6	3.9
**Polypharmacy, % Yes**	41.1	40.0
**Drug-Drug Interaction, % Yes**	12.1	11.5
**Potentially Inappropriate Medication, % Yes**	37.5	37.6

Supplemental Table 2. Proportion of respondents experiencing death by cohort entry cycle

Entered Cohort	Unweighted Deaths, N	Weighted Proportion Died
1999-2000	587	0.795
2001-2002	553	0.665
2003-2004	572	0.630
2005-2006	428	0.515
2007-2008	514	0.485
2009-2010	367	0.342
2011-2012	254	0.284
2013-2014	199	0.198
2015-2016	117	0.098

*All followed through 2019

Supplemental Table 3. Average marginal effects on probability of medication regimen with polypharmacy, potentially inappropriate medications use, or a drug-drug interaction

		Probability of polypharmacy		Probability of potentially inappropriate medication		Probability of drug-drug interaction	
		Baseline Probability 40.01%		*Baseline Probability* 36.80%		*Baseline Probability* 11.50%	
		Average marginal effect	*P*	*Average marginal effect*	*P*	*Average marginal effect*	*P*
**Age (per year)**		0.14% (-0.11 - 0.39)	0.26	0.03% (-0.24 - 0.30)	0.82	0.04% (-0.14 - 0.23)	0.64
**Sex (Ref =Female)**	*Male*	-7.19% (-10.00 - -4.29)	<0.01	-6.50% (-9.52 - -3.40)	<0.01	-0.54% (-2.46 - 1.38)	0.58
**Race (Ref=NH White)**	*Hispanic*	-1.59% (-6.21 - 3.06)	0.50	-2.70% (-7.04 - 1.64)	0.22	-0.37% (-2.81 - 2.08)	0.77
	NH Black	-1.26% (-5.16 - 2.66)	0.53	-6.45% (-9.96 - -2.94)	<0.01	0.68% (-2.11 - 3.47)	0.63
	Other	0.26% (-7.46 - 7.98)	0.95	-0.92% (-8.77 - 6.93)	0.82	1.84% (-2.62 - 6.29)	0.42
**Education (Ref =College Graduate)**	*Less than High School*	1.42% (-2.65 - 5.47)	0.49	2.05% (-2.59 - 6.61)	0.38	2.02% (-0.65 - 4.72)	0.14
	High School Graduate	1.24% (-3.32 - 5.76)	0.59	6.77% (1.96 - 11.33)	<0.01	-0.15% (-2.77 - 2.47)	0.91
	Some College	2.94% (-0.98 - 6.81)	0.14	3.47% (-1.05 - 7.86)	0.13	0.60% (-2.13 - 3.33)	0.67
**Income-to-Poverty Ratio (per unit)**		-0.18% (-1.41 - 1.05)	0.77	0.83% (-0.19 - 1.82)	0.11	-0.07% (-0.76 - 0.61)	0.83
**Cardiovascular Disease (Ref=No)**	*Yes*	19.61% (15.65 - 23.22)	<0.01	6.85% (2.80 - 10.76)	<0.01	4.96% (2.52 - 7.38)	<0.01
**Congestive Heart Failure (Ref=No)**	*Yes*	22.15% (16.43 - 27.50)	<0.01	11.09% (5.62 - 16.36)	<0.01	7.02% (2.82 - 11.23)	<0.01
**Chronic Obstructive Pulmonary Disease (Ref=No)**	*Yes*	17.86% (13.16 - 22.18)	<0.01	7.73% (2.76 - 12.56)	<0.01	2.27% (-0.92 - 5.47)	0.17
**Liver Disease (Ref=No)**	*Yes*	11.21% (3.76 - 18.44)	0.03	5.70% (-2.83 - 14.13)	0.19	0.49% (-4.14 - 5.12)	0.84

**History of Cancer (Ref=No)**	*Yes*	5.05% (2.11 - 7.92)	0.01	2.94% (0.21 - 5.60)	0.03	2.30% (0.25 - 4.38)	0.03
**Smoking Status (Ref=Never Smoker)**	*Current Smoker*	-0.77% (-4.80 - 3.27)	0.71	0.77% (-4.54 - 6.06)	0.78	-2.17% (-5.07 - 0.71)	0.14
	Former Smoker	7.17% (3.94 - 10.28)	<0.01	4.63% (1.25 - 7.90)	0.01	3.49% (1.42 - 5.60)	<0.01
**Alcoholic Drinks (per day)**		-1.99% (-3.66 - -0.28)	0.02	-1.10% (-2.94 - 0.76)	0.24	-0.31% (-1.61 - 0.99)	0.64
**Anemia (Ref=No)**	*Yes*	12.14% (3.92 - 20.12)	<0.01	10.29% (1.28 - 19.12)	0.02	2.55% (-2.89 - 7.99)	0.36
**AST Elevated (Ref=No)**	*Yes*	9.37% (-1.25 - 19.81)	0.08	3.27% (-7.63 - 14.11)	0.56	0.47% (-7.12 - 8.06)	0.90
**ALT Elevated (Ref=No)**	*Yes*	-10.80% (-23.44 - 2.04)	0.10	-12.63% (-25.47 - 0.57)	0.06	0.33% (-9.41 - 10.08)	0.95
**Systolic Blood Pressure (per mmHg >130)**		-0.09% (-0.17 - -0.00)	0.04	0.03% (-0.05 - 0.11)	0.45	0.01% (-0.03 - 0.06)	0.62
**Diastolic Blood Pressure (per mmHg >90)**		-0.93% (-1.85 - 0.02)	0.05	-1.42% (-2.43 - -0.38)	<0.01	-0.33% (-0.98 - 0.33)	0.33
**BMI (Ref=Abnormal)**	*Normal*	-9.17% (-12.04 - -6.11)	<0.01	-4.44% (-7.57 - -1.20)	<0.01	-1.46% (-3.37 - 0.45)	0.14
**A1c (per unit increase)**		9.98% (6.75 - 13.02)	<0.01	3.77% (1.57 - 5.89)	<0.01	0.90% (-0.31 - 2.11)	0.15
**eGFR (per unit increase)**		-0.36% (-0.43 - -0.28)	<0.01	-0.09% (-0.17 - -0.01)	0.03	-0.08% (-0.13 - -0.03)	<0.01

A. Average marginal effects represent the estimated change in the probability associated with a one-unit change in a predictor variable, while holding other variables constant. For example, holding all other variables constant, being male is associated with a 7.19% lower probability of experiencing polypharmacy compared with being female. B. Abbreviations: Ref=Reference category for model, NH=Non-Hispanic, AST =aspartate aminotransferase, ALT= alanine aminotransferase, BMI=body mass index in kg/m^2, a1c= glycated hemoglobin as a percentage, eGFR=estimated glomerular filtration rate in mL/min/1.73m

Supplemental Figure 2. Event-free survival by polypharmacy status by follow-up time

A. Complete follow-up time

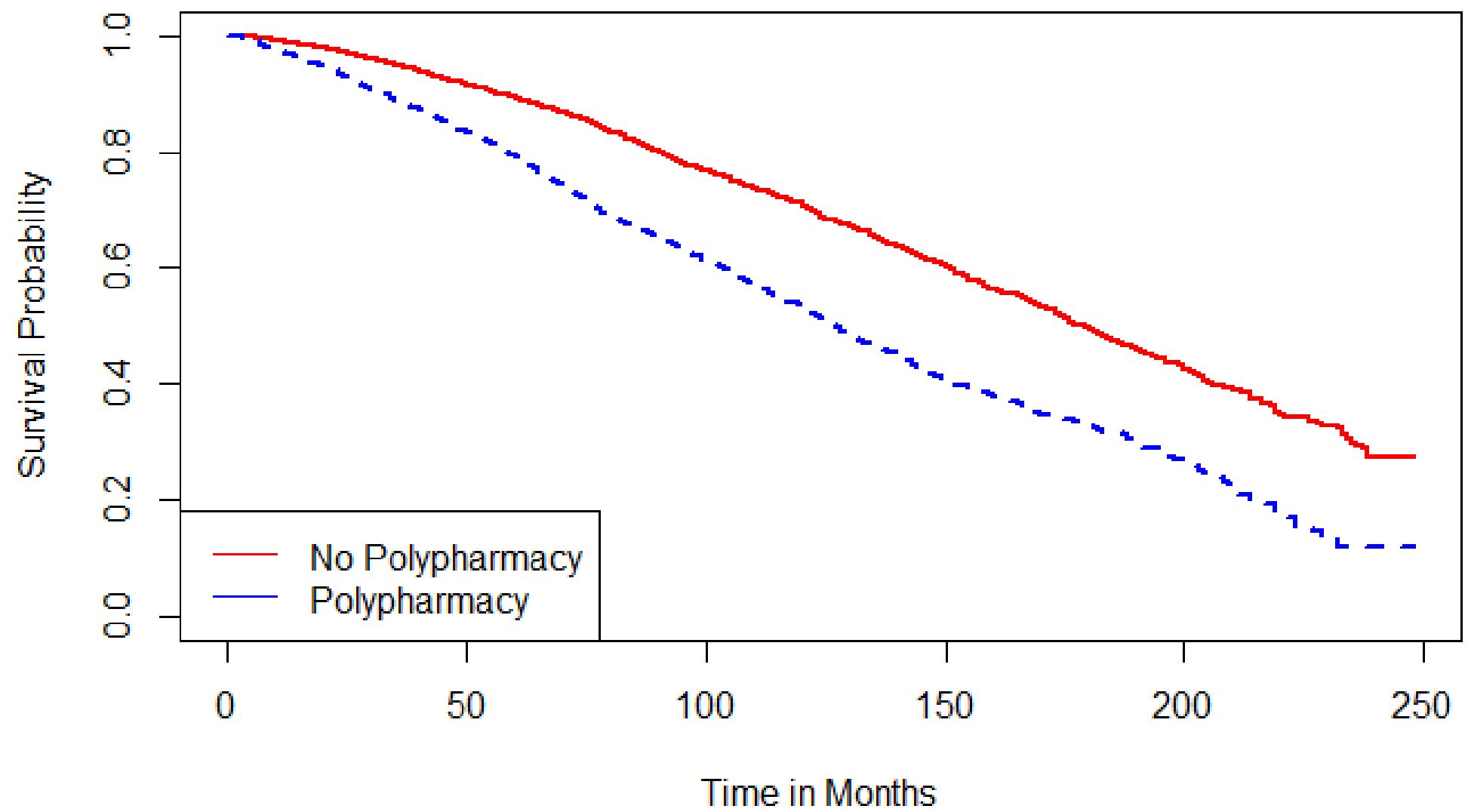

*Model 3: hazard ratio 1.38, 95%CI 1.26-1.51

B. Limited to first 2 years

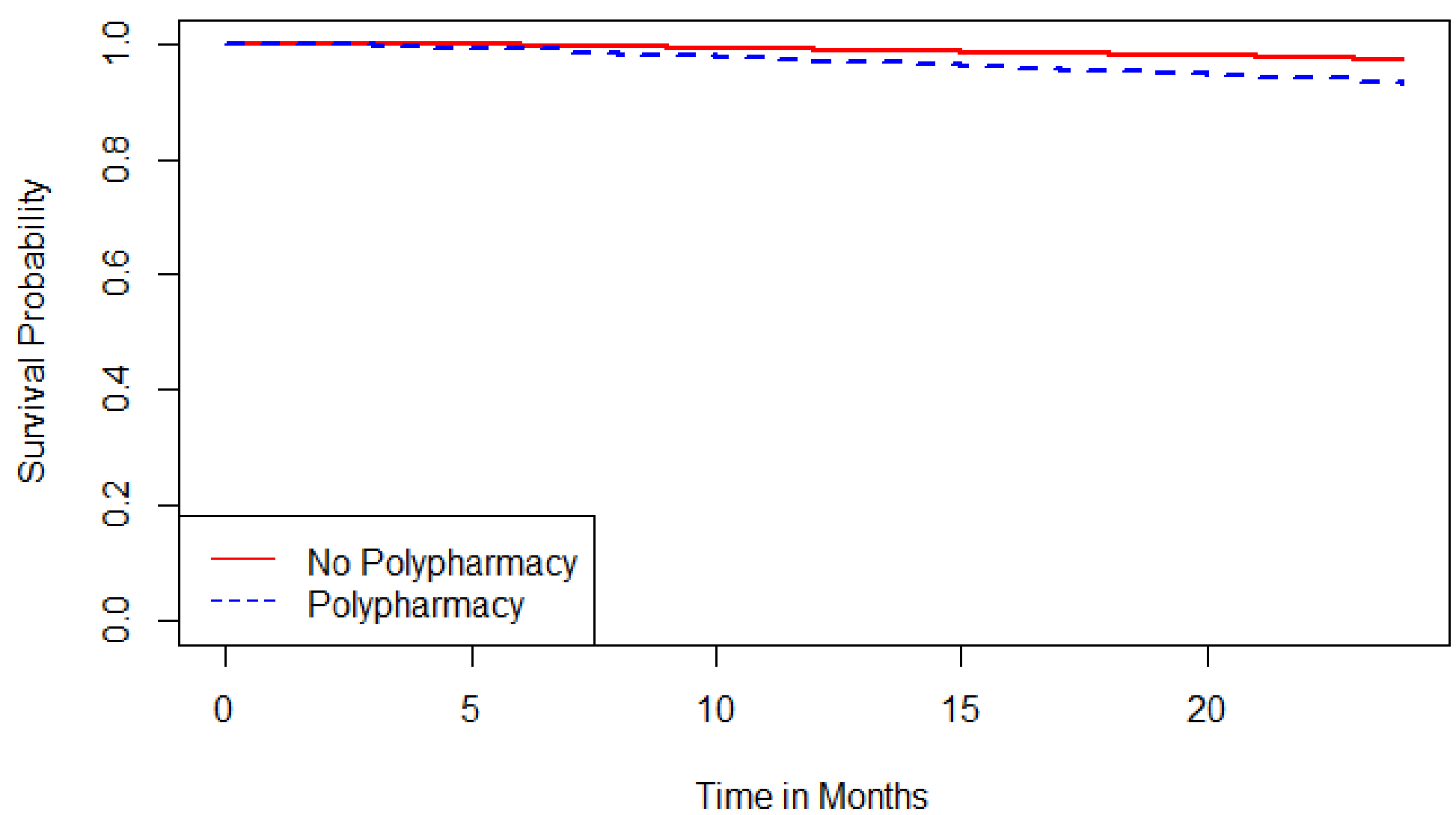

*Model 3: hazard ratio 1.53, 95%CI 1.14-2.06

Supplemental Figure 3. Event-free survival by potentially inappropriate medication use status by follow-up time

A. Complete follow-up time

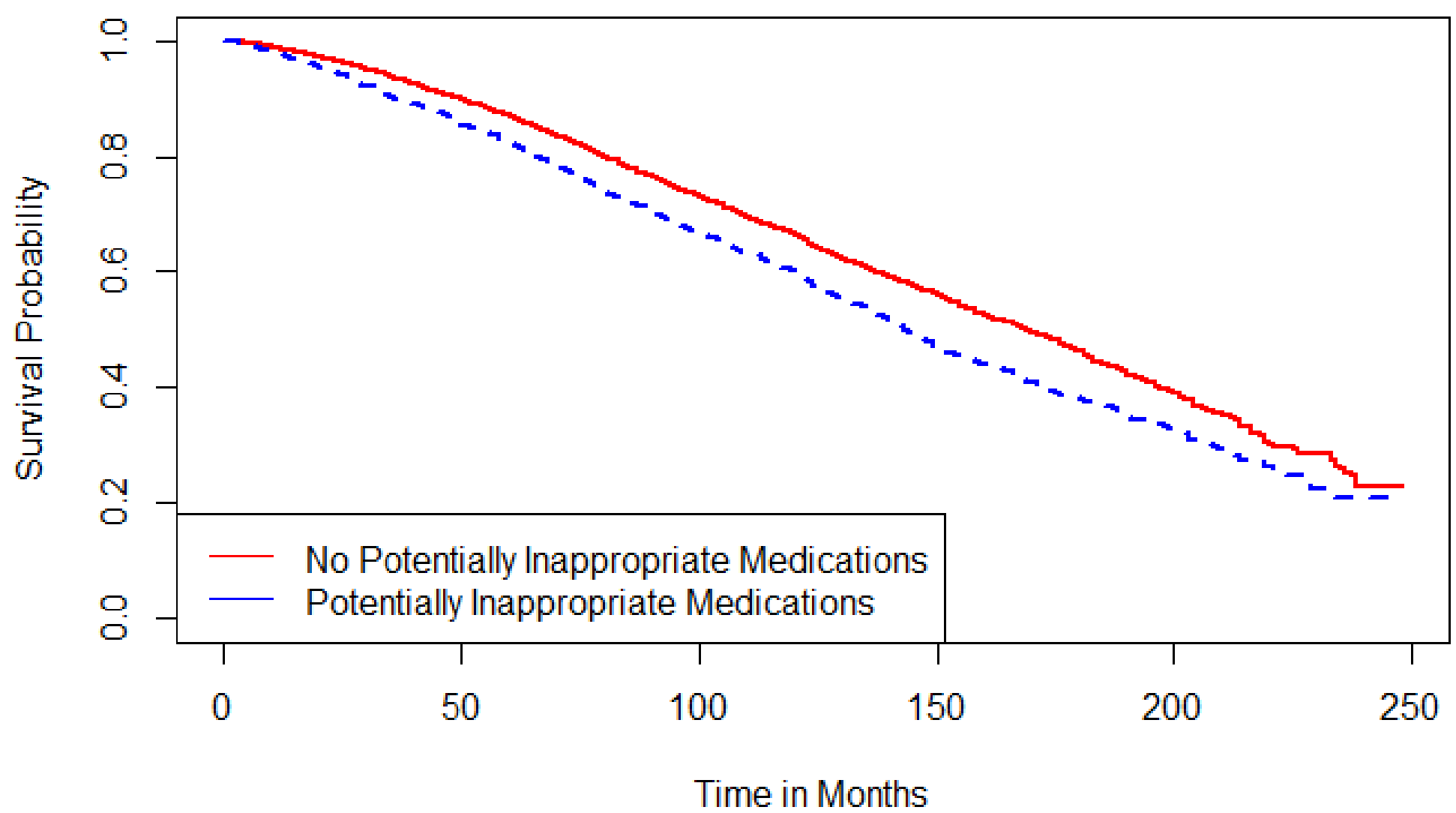

*Model 3: hazard ratio 1.05, 95%CI 0.97-1.14)

B. Limited to first 2 years

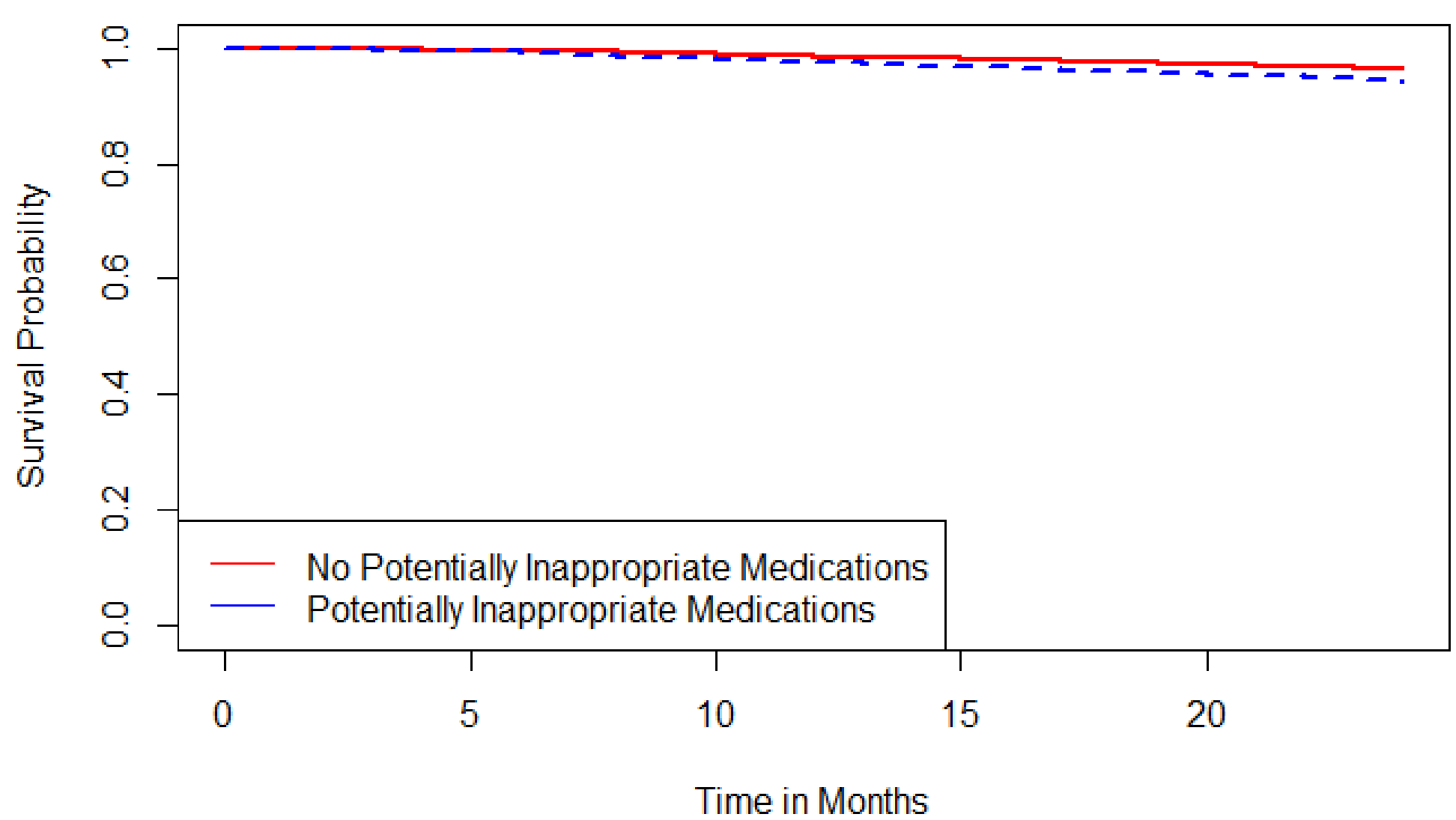

*Model 3: hazard ratio 1.10, 95%CI 0.88-1.39

Supplemental Figure 4. Event-free survival by drug-drug interaction status by follow-up time

A. Complete follow-up time

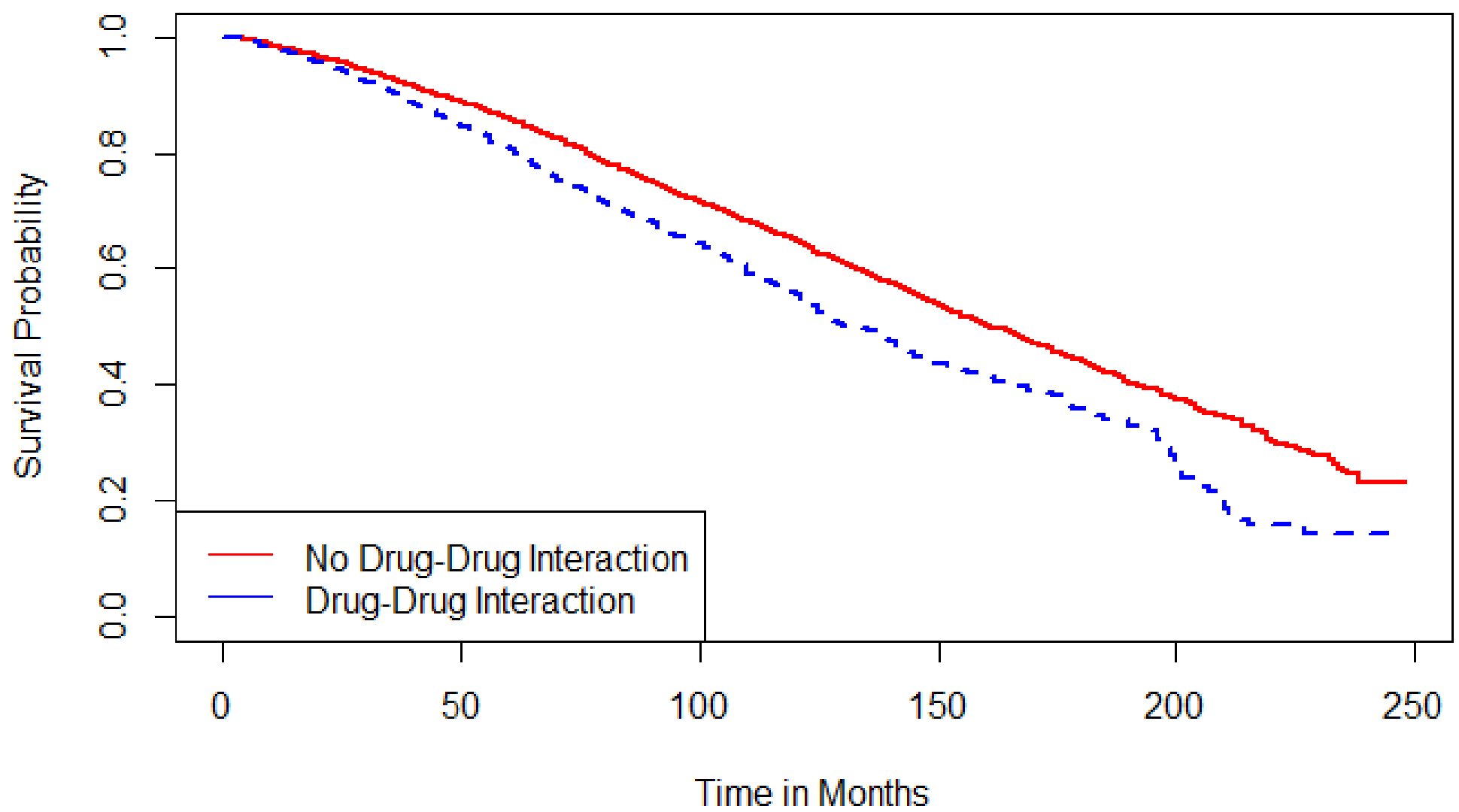

*Model 3: hazard ratio 1.02, 95%CI 0.91-1.16

B. Limited to first 2 years

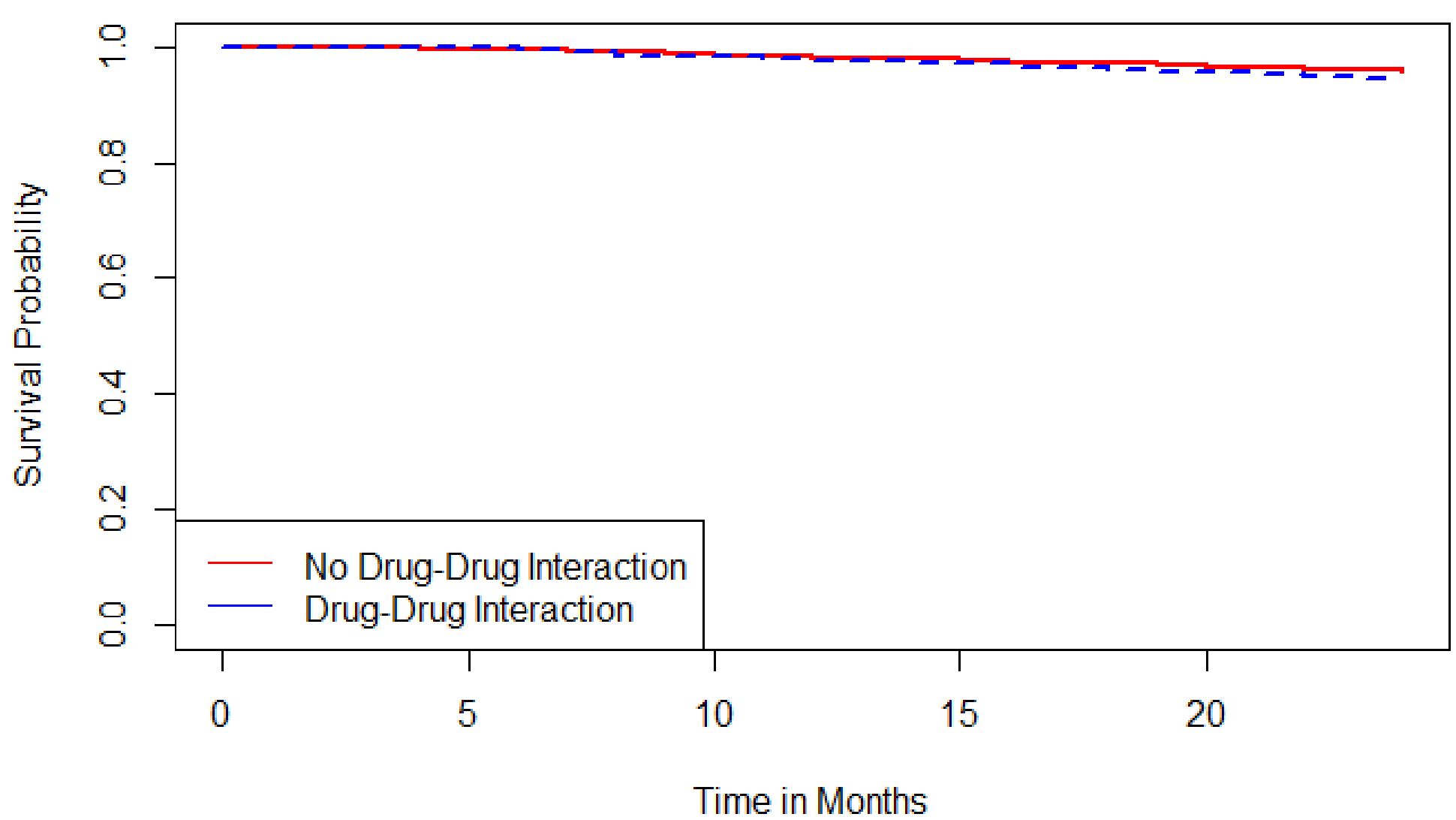

*Model 3: hazard ratio 0.82, 95%CI 0.60-1.13
